# Development and Characterization of Crackers Substitution of Wheat Flour With Jellyfish

**DOI:** 10.3389/fnut.2021.772220

**Published:** 2021-12-06

**Authors:** Suchada Maisont, Wisutthana Samutsri, Wuttichai Phae-ngam, Pichet Limsuwan

**Affiliations:** ^1^Department of Food Science and Technology, Faculty of Science and Technology, Phranakhon Rajabhat University, Bangkok, Thailand; ^2^Physics Program, Faculty of Science and Technology, Phranakhon Rajabhat University, Bangkok, Thailand; ^3^Department of Physics, Faculty of Science, King Mongkut's Institute of Technology Ladkrabang, Bangkok, Thailand

**Keywords:** edible jellyfish, *Lobonema smithii*, cracker, textural property, sensory evaluation, shelf life

## Abstract

The objective of this study was to investigate the possibility of using jellyfish (*Lobonema smithii*) for the production of nutritionally improved crackers. In this study, ground jellyfish were incorporated into different levels (20, 30, and 40%) to replace wheat flour in cracker formula. Physicochemical characteristics (linear expansion, hardness, and color) and sensory quality of the developed crackers were examined and compared with control crackers. The crackers with jellyfish were found significantly darker, with more brittleness, and less consumer accepted than the control samples (*p* < 0.05). Moisture content, *a*_w_, and thiobarbituric acid reactive substances (TBARS)-values of jellyfish crackers increased while the hardness of the jellyfish crackers decreased with increasing the storage time for both crackers stored at 35 and 45°C. The substitution of wheat flour with jellyfish led to high protein content in the crackers. The cracker with 30% of jellyfish gained characteristics of cracker, liking scores, as well as the subjective quality of the final product and had good physical and chemical conditions, being able to be consumed for 12 weeks stored at 35°C.

## Introduction

Thailand is one of the world's largest producers of edible jellyfish among Asian countries besides China, Indonesia, India, Vietnam, Malaysia, and the Philippines (1). Jellyfish are caught along the coastal areas of 16 provinces in the Pacific Ocean (Gulf of Thailand) and 6 provinces in the Indian Ocean (Andaman Sea). A recent study by the Marine and Coastal Resources, Thailand, during the year 2010–2015 reported that most edible jellyfish found in both seas belong to the species *Lobonema smithii* and *Rhopilema hispidum* (2).

After the jellyfish were caught in the sea, they were separated into the umbrella and oral arms for processing. The jellyfish were first processed in salt and alum for approximately 2 weeks. The salted jellyfish are left in the brine for 3–4 days and dried at room temperature for 2 days. Then, the jellyfish is packaged and stored in dried salt. The above processing jellyfish in the form of salted product is a traditional method for preservation and exportation.

The nutritional composition in various species of edible jellyfish has been widely studied (3–8). Fresh jellyfish contains high water content, easily decomposes, and lyses into inorganic constituents, including Zn, Co, Ni, Ba, Mn, Fe, Mg, Ca, Cu, Al, Sr, Mo, Cr, Cd, Pb, Si, V, Ti, Na, K, Li, and Rb (9). In general, all jellyfish have low caloric values (3), high protein (collagen) (10–12), and high amino acid (13, 14). Edible jellyfish are rich in protein and minerals while low in fats and calories. Collagen was found to be the major protein in edible jellyfish. Having good protein quality and low calories, edible jellyfish is an appealing source of nutritive ingredients for the development of oral formulations, nutricosmetics, and functional food.

Crackers are a type of snack that have long been popular worldwide for over 150 years. Nowadays, they are also popular snack foods in Thailand. Due to their convenience and stable shelf-life, and include broad varieties such as soda crackers or saltines, chemically leavened crackers, and savory (flavored) crackers. Soda crackers are made using a sponge and dough method and are fermented for up to 24 h by yeast, which contributes to their unique texture and flavor attributes (15). Chemically leavened crackers are similar to fermented crackers except for their method of leavening, which is sodium bicarbonate with an acidifying salt. Since these crackers do not go through gluten development and fermentation, there is no requirement for blending hard wheat with higher levels of stronger gluten (16). Savory crackers are most often produced using the fermentation method, during which flavorings such as cheeses, herbs, or spices may be added (15). More than ever, consumers are seeking broader and more nutritive functions from their snacks as they become a bigger part of their daily diet. The main ingredient of crackers is flour so that crackers have a low protein content. With the increasing demand of health-oriented products, crackers with healthy ingredients such as protein and dietary fiber have gained increasing interest. In the past decade, wheat flour was substituted with various healthy ingredients from agricultural products (17–27) and fishes (28–34).

As far as we know, no information has been published on jellyfish crackers, that is the wheat flour was substituted with jellyfish. Therefore, the development and characterization of jellyfish crackers with different jellyfish content were studied. The purpose of this work was to investigate the suitability of using jellyfish for the production of savory crackers concerning the characteristics and subjective quality of the final product.

## Materials and Methods

### Materials

Jellyfish (*L. smithii*) were obtained from Chockdee Sea Product Co. Ltd., Samutsongkhram Province, Thailand. In this work, rings of muscle around the umbrella and the digestive and reproductive systems of salted jellyfish, a by-product of the processing of salted jellyfish product were used. Wheat flour was purchased from United Flour Co. (Samutprakan Province, Thailand). Instant yeast was purchased from Saf-instant (Wisconsin, United States). Other baking ingredients including shortening, refined sugar, salt, baking powder, and vegetable oil were purchased from a local supermarket in Bangkok, Thailand.

### Preparation of Jellyfish

The salted jellyfish was washed with water for about three times until the salt content was about 2–3%. Then, they were soaked in the water for 10 min and drained.

After draining, to reduce some excess water from the samples and to produce half flavor due to smoking and burning of the surface samples, they were roasted for about 10 min until moisture content was approximately reduced to 63–65% from 91–92%. The roasted samples were blended in a blender for 2–3 min to obtain the ground jellyfish. It was kept in polyethylene bags and stored at 4–10°C until used. Moisture, protein, and fat contents of ground jellyfish were analyzed according to the AOAC method (35).

### Preparation of Raw Cracker Sample

The cracker formula used in the study was based on the two-stage mixing process, known as sponge and dough method (36). The ingredients of sponge and dough are given in Table 1. Wheat flour content in the dough process was partially replaced with ground jellyfish at the concentrations of 20, 30, and 40% (w/w) of total wheat.

**Table 1:** Basic cracker formula, modified from Maisont and Khucharoenpaisan (37).

	**Ingredients**	**Quantity (g)**
**Sponge**	Wheat flour	385
	Water	200
	Yeast	4
	Sugar	150
**Dough**	Wheat flour	615
	Shortening	200
	Glucose syrup	20
	Vegetable oil	6
	Milk powder	50
	Baking powder	7
	Salt	18
	Water	106

From Table 1, the wheat flour was mixed with other ingredients for the sponge formation, as follows: wheat flour, water, yeast, and refined sugar, kneaded in the kneading machine (Kittiwattana, Thailand) for 15 min, and left in the dough incubator at 35°C for 2 h to allow it to prove, then, the sponge or fermented dough was obtained.

The dough process was prepared based on the standard method without any modification by mixing of ingredients (Table 1). In this work, the wheat flour contents of 615, 415, 315, and 215 g were varied and the ground jellyfish contents of 0, 200, 300, and 400 g were added, respectively. After that, the mixing was kneaded in a kneading machine (Kittiwattana, Thailand) for 5 min and the dough was obtained.

All the four final dough samples were prepared by kneading the sponge and the dough for 5 min, then, allowed to rest in a dough incubator at 35°C for 30 min. The final dough was subsequently sheeted using a pastry dough sheeter (Kittiwattana, Thailand) with a 0.7–1.0 mm thickness and size of 20 × 30 cm and then shaped using a mold of the size 4 × 5 cm. After that, the 90–110 pieces of each batch were baked at 180°C for 15 min and then they were then left at room temperature for cooling. Finally, the jellyfish cracker products were obtained. Therefore, in this work, jellyfish cracker samples substituted wheat flour with ground jellyfish of 0, 20, 30, and 40% were fabricated and denoted as JC_0_, JC_20_, JC_30_, and JC_40_, respectively. The crackers without any ground jellyfish were used as control sample.

### Physicochemical Measurement

#### Linear Expansion Measurement

The thickness of four points as the result of a cross mark of each slice of jellyfish crackers before and after baking at 35°C was measured with a Vernier caliper and replicated 10 times. The linear expansion of the puffed cracker was calculated from the difference in the average value of thickness before and after baking.

#### Color Measurement

The color of jellyfish crackers was measured using a colorimeter (Minolta, model CR-10, Japan) in CIE chromaticity coordinates L^*^, a^*^, and b^*^, where L^*^ describes lightness on a scale of 0–100 (where 0 = black and 100 = white), a^*^ describes intensity in the red–green axis (positive a^*^-values indicate red undertone, negative a^*^-values indicate green undertone), and b^*^ describes intensity in the yellow–blue axis (positive b^*^-values mean yellow undertone, negative b^*^-values mean blue undertone).

#### Textural Measurement

The textural property of jellyfish crackers was measured in terms of hardness or brittle property using a TA.XT plus texture analyzer (Stable Micro Systems, Surrey, United Kingdom). A knight blade of 3 mm thick with a flat end probe for the puncturing test was used. The settings for texture measurement of jellyfish crackers were performed to measure the force (*N*) in compression up to 25 mm distance, with 5 mm/s pretest speed, 1 mm/s test speed, 10 mm/s post-test speed, and trigger auto force set to 20 g. The peak value of the fracture force (maximum), at the point when the cracker would break into two major pieces was recorded as hardness. The hardness evolution was assessed on 10 jellyfish crackers for each trial. The texture expert program version 6.1.1.0 was used for data analysis.

#### Water Activity Measurement

Water activity (*a*_w_) was measured using a water activity meter (Aqua Lab, Series 3 WA, United States). Water activity represents the ratio of the water vapor pressure of the food to the water vapor pressure of the pure water under the same conditions and is expressed as a fraction. The water activity scale extends from 0.0 (dry matter) to 1.0 (pure water). Approximately 2 g of ground cracker sample was placed in the water activity tray and *a*_w_ was recorded.

### Sensory Evaluation

Sensory evaluation of the cracker was performed with 30 untrained panelists in a sensory laboratory with separate booth designs. Samples were placed on white polystyrene plates labeled with three-digit random numbers and presented to panelists in a randomized order. Between each sensory test, panelists were given water to rinse their palate, to reduce sensory fatigue. Panelists evaluated the samples for appearance, color, taste, salted odor, and overall liking using a seven-point hedonic scale, where 1 represented extremely dislike and 7 the opposite extreme.

### Analysis of the Final Product

Physicochemical properties and sensory results play an important role in the characteristics of crackers. Therefore, the suitability of jellyfish content for production of crackers concerning the characteristics and subjective quality of the final product was selected for this study.

#### Proximate Analysis

The proximate composition of the sample, that is, moisture, ash, protein, fat, crude fiber, and carbohydrate, was determined according to the method established by AOAC (35). The moisture content was determined using the oven drying method at 105°C up to a constant weight. The ash content was conducted in a muffle furnace at 550°C for 3 h until a white or light gray ash was obtained. Protein content was determined using the Kjeldahl method with Nx6.25. The Soxhlet extraction method was used for the determination of fat content. The crude fiber was analyzed by the loss on ignition of the dried residue remaining after digestion of the sample and determined by weight difference. Carbohydrate content was calculated by difference. The result was reported as g/100 g dry matter.

#### Shelf Life Study

Analysis of the shelf life of the chosen cracker was carried out once in 2 weeks for 12 weeks. The samples were packed in laminated aluminum foil bags and stored in two controlled conditions at temperatures of 35 and 45°C, respectively. Storage stability or the shelf life of products could be defined as maintenance of the characteristics associated with sensory acceptability. Thus, the properties of the final product related to texture changes and rancidity, including moisture content, *a*_w_, texture property, and thiobarbituric acid reactive substances (TBARS)-value were observed. Thiobarbituric acid reactive substances value is a measure of the formation of secondary oxidation products, for example, carbonyls, being responsible for the sensory impact of lipid oxidation. It was measured using the assay measure of malondialdehyde (MDA). The MDA reacts with thiobarbituric acid (TBA) forming a pink chromogen (TBARS), which is measured spectrophotometrically at 532–535 nm. A linear regression curve of standard of 1,1,3,3-tetraethoxypropane (TEP) vs. the relative light unit for each standard was plotted. The TBARS as the milligram of MDA per 1 kg of sample was calculated using the relative light unit obtained from the sample and equation generated by the standard curve.

### Statistical Analysis

Crackers were prepared in triplicate batches; mean values and standard deviations were calculated and all results were expressed as mean ± SD. Analysis of variance (one-way ANOVA) was carried out using SPSS (IBM SPSS statistics version 16). Duncan's new multiple range test was used to find out the statistically significant differences of mean values between treatments at the level at *P* ≤ 0.05.

## Results and Discussion

### Characteristics of Raw Cracker Sample

**Figure 1:**
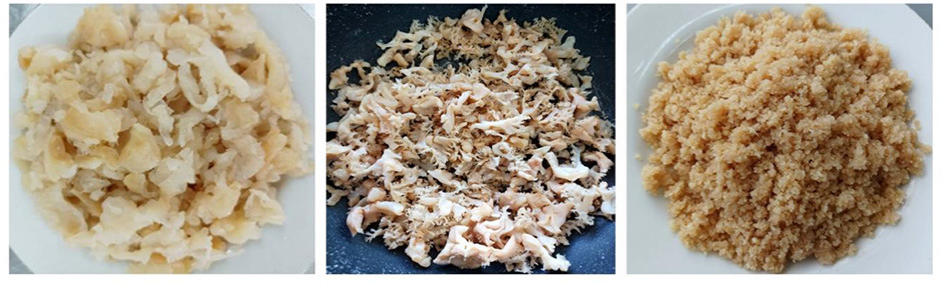
Characteristics of jellyfish, roasted jellyfish, and ground jellyfish.

The color characteristics of jellyfish, roasted jellyfish, and ground jellyfish were presented in Figure 1. The color of ground salted jellyfish was light brown according to L^*^-value, was a high value of 55.10 while the a^*^-value and the b^*^-value were low values of 10.76 and 12.40, respectively.

Roasting reduced the moisture content of the salted jellyfish from 91–92% to 63–65%. The protein content of ground salted jellyfish was 6.47%. In our study, the content of protein was higher than those reported by Kromfang et al. (10). Generally, all jellyfish owned low calorific values and negligible fat contents (3). The fat content of ground salted jellyfish in this study was 0.38%.

### Physicochemical Characteristics of Jellyfish Crackers

**Figure 2:**
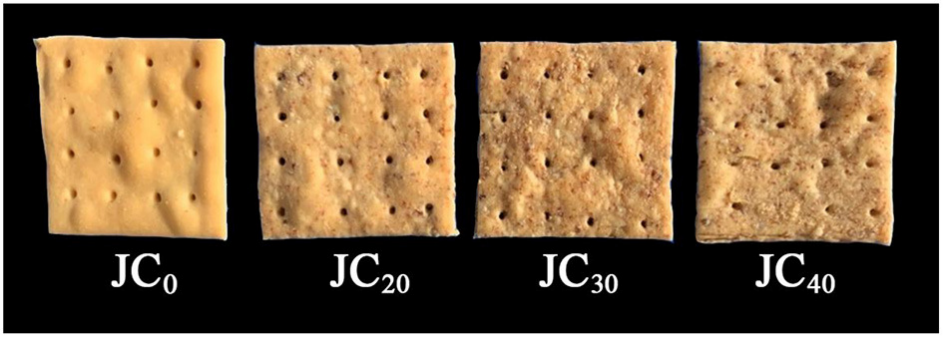
Characteristics of cracker samples.

Figure 2 shows the photograph of cracker samples substituted with different jellyfish of 0, 20, 30, and 40% of wheat flour (w/w) as denoted by JC_0_, JC_20_, JC_30_, and JC_40_, respectively. The results of the measurements of moisture content, water activity, linear expansion, hardness, and color of jellyfish crackers are given in Tables 2, 3.

**Table 2:** Moisture content and water activity of four jellyfish cracker samples.

**Cracker sample**	**Jellyfish content (%)**	Moisture content^*^ (%)	${\text{a}}_{\text{w}}^{*}$
JC_0_	0	2.55 ± 0.49^a^	0.27 ± 0.01^a^
JC_20_	20	2.19 ± 0.75^a^	0.18 ± 0.01^b^
JC_30_	30	1.15 ± 0.76^ab^	0.18 ± 0.01^b^
JC_40_	40	0.97 ± 0.20^b^	0.16 ± 0.01^c^

* *Mean ± standard deviation values in the same column for each sample followed by different letters are significantly different (p ≤ 0.05)*.

**Table 3:** Linear expansion, hardness, and color of jellyfish crackers.

**Cracker sample**	**Jellyfish content (%)**	**Linear expansion (mm)**	**Hardness (*N*)**	**Color**		
				*L* ^*^	*a* ^*^	*b* ^*^
JC_0_	0	1.89 ± 0.07^a^	10.76 ± 1.43^a^	56.44 ± 1.82^a^	10.89 ± 0.88^b^	28.91 ± 0.44^a^
JC_20_	20	1.75 ± 0.46^a^	6.72 ± 1.27^b^	56.11 ± 0.81^a^	11.70 ± 0.17^b^	27.88 ± 0.31^b^
JC_30_	30	1.57 ± 0.20^ab^	5.24 ± 0.68^ab^	54.43 ± 1.11^ab^	11.31 ± 0.18^b^	27.14 ± 0.51^b^
JC_40_	40	1.39 ± 0.26^b^	5.14 ± 0.86^c^	52.87 ± 1.97^b^	12.89 ± 0.16^a^	26.35 ± 0.34^c^

* *Mean ± standard deviation values in the same column for each sample followed by different letters are significantly different (p ≤ 0.05)*.

From Table 2, the moisture content and *a*_w_ ranged from 0.97 to 2.55% and 0.16 to 0.27, respectively. It is seen that the moisture content and *a*_w_ decreased with increasing the substituted jellyfish. The *a*_w_ of crackers without jellyfish (JC_0_) were statistically significant at *p* ≤ 0.05 compared with those of the substituted jellyfish crackers (JC_20_, JC_30_, and JC_40_). Similar results were observed by Prapasuwannakul (38) and Asikin et al. (39), who incorporated fish bone powder in different levels to replace flour in the snack cracker. As a dried product, jellyfish crackers are expected to have low moisture. Control of moisture is necessary to optimize the quality of crackers (40). During gelatinization, starch granules in suspension absorb water, swell, and eventually solubilize (41). The ability to absorb water is a very important property of all flours and starches used in food preparations. Excessive water can also cause a decrease in expansion, resulting in a thinner and less crisp snack or in a very soft dough, which is difficult to shape (40). The decrease in moisture content and *a*_w_ satisfied with the decrease of wheat flour content. This could be due to the insufficient starch content for absorption of water for gelatinization, less water was entrapped in the starch gel. Insufficient water can lead to incomplete gelatinization of starch during the production process (42).

From Table 3, the linear expansion and hardness ranged from 1.39 to 1.89 mm and 5.14 to 10.76 N, respectively. It is seen that the linear expansion and hardness decreased with increasing the substituted jellyfish. The linear expansion and hardness of crackers JC_0_ without jellyfish were statistically significantly different (*p* ≤ 0.05) compared with those of crackers JC_40_. The decrease in the degree of linear expansion is related to the increase in the proportion of jellyfish. It would appear that the substituted jellyfish interacted with the starch granules in a way that inhibited expansion. This might be caused by the starch in the unexpanded portion that has not been fully gelatinized, the insufficient starch content for absorption of water for gelatinization. In our experiment, the addition of jellyfish to wheat flour lowered the amount of starch in the mixtures, causing poor water absorption and thus reduced volume and linear expansion of the crackers. The results are in agreement with findings by Prapasuwannakul (38). During gelatinization, starch granules in suspension absorb water, swell, and eventually solubilize (41). Starch granules that are fully gelatinized will result in better rupture of the starch cells during the gelatinization process. The degree of expansion of the product is influenced by the amylose to amylopectin ratio of the flour. At least 50% or more of amylopectin and 5–20% of amylose are required for a good quality cracker (43).

Because less water was trapped in the network of starch gel, the expansion decreased. As shown in Table 3, the less the degree of the expansion of the crackers, the lesser the air cells were formed and trapped; consequently, the lower the hardness of the cracker. High porosity means a high volumetric content of the air that does not contribute to hardness (32).

For color measurement, the L^*^-value slightly decreased when the substituted jellyfish was increased, indicated a darker color of the cracker was subsidized from jellyfish. This result was corresponded with Nurul et al. (40) which stated the fish contains some pigments that contribute to the color of the product. Since the different protein content is responsible for the degree of loss of the L^*^-value, the loss of the L^*^-value is higher in the sample with an increase in the ratio of fish in the product. Furthermore, the increase of a^*^-value and the decrease of b^*^-value with increasing the substituted jellyfish confirm the increase in cracker darkness, the resulting crackers are light brown. Kaewmanee et al. (32) found that lightness and redness tended to decrease with the ratio of fish meat to tapioca flour, whereas yellowness tended to decrease. Generally, the factors affecting the color of crackers include the amount and type of starch used, as well as the amount and type of protein added. In addition Millar et al. (44) suggested that the darker color of the crackers containing pulse flours can be partly attributed to Maillard browning from reactions between amino acids and reducing sugars at a high temperature.

### Sensory Analysis Results

The sensory analysis on appearance, color, odor, taste, crispiness, and overall acceptability for crackers JC_0_, JC_20_, JC_30_, and JC_40_ is presented in Table 4.

**Table 4:** Sensory analysis for the overall acceptance of jellyfish crackers^*^.

**Sensory attributes**	**Jellyfish substitution (%)**			
	**0**	**20**	**30**	**40**
Appearance	6.66 ± 0.47^a^	6.43 ± 0.50^ab^	6.36 ± 0.49^b^	6.26 ± 0.44^b^
Color	6.53 ± 0.50^a^	6.33 ± 0.54^ab^	6.46 ± 0.50^a^	6.10 ± 0.54^b^
Odor	6.23 ± 0.43^ab^	6.40 ± 0.49^a^	6.36 ± 0.49^a^	6.13 ± 0.34^b^
Taste	6.23 ± 0.43^ab^	6.03 ± 0.55^b^	6.33 ± 0.47^a^	5.93 ± 0.52^c^
Crispiness	6.30 ± 0.46^a^	6.23 ± 0.50^a^	6.36 ± 0.49^a^	5.90 ± 0.60^b^
Overall acceptability	6.30 ± 0.46^ab^	6.10 ± 0.30^bc^	6.36 ± 0.49^a^	6.06 ± 0.36^c^

* *Mean ± standard deviation values in the same column for each sample followed by different letters are significantly different (p ≤ 0.05)*.

There were no significant differences (*p* > 0.05) in terms of color, odor, taste, crispiness, and overall acceptability of JC_0_, JC_20_, and JC_30_; the scores of the jellyfish crackers ranged between 6.03 (like) and 6.66 (like very much) (Table 4). However, there were significant (*p* < 0.05) differences in terms of the appearance of JC_0_ and JC_20_ compared to JC_30_ and JC_40_. Moreover, the lowest score in color, odor, taste, crispiness, and overall acceptability was found with crackers enriched with substituted jellyfish at levels of 40%. This result indicated that a negative effect of some desirable characteristics of the cracker was noticed with an increase in the jellyfish content, since the characteristics of jellyfish in the present study are salted jellyfish and quite a gritty paste. Consequently, the panelists could detect salted odor and sandiness-like mouth feel in the sample. Thus, this is the reason that the crackers with the substitution of jellyfish at levels of 40% obtained a lower organoleptic score. A similar observation has been documented by Fong-in et al. (45), who found that the addition of fish bone (at levels 10, 20, 30, and 40%) in cashew nut cookies decreased the sensorial acceptance of them when compared to the control samples.

Based on the sensory results, JC_20_ and JC_30_ were comparable in liking scores. The organoleptic score of jellyfish cracker is taken into consideration and it reflects the degree of liking or disliking for the product, which in turn is used to predict acceptability. However, sensorial result is not the only parameter to be analyzed; other analyses such as physical, chemical, and especially functional properties also play an important role. Therefore, only the cracker JC_30_ was considered for further study. The cracker JC_30_ samples were referred to the sample of zero week (week 0) and proximate composition of crackers was determined, and the shelf life study was carried out.

### Characteristics of the Final Product

#### Proximate Composition

Crackers JC_30_ samples were analyzed for moisture content (standard oven dry method), protein content (Kjeldahl method), fat content (Soxhlet method), fiber content (Weende method), ash, and carbohydrate contents according to the AOAC method (35). The results of the measurements of moisture, fat, protein, fiber, ash, and carbohydrate content in cracker JC_30_ were 1.48 ± 0.5, 8.25 ± 0.06, 13.09 ± 0.14, 0.41 ± 0.03, 2.89 ± 0.66, and 73.88 ± 0.74, respectively.

#### Shelf Life

**Figure 3:**
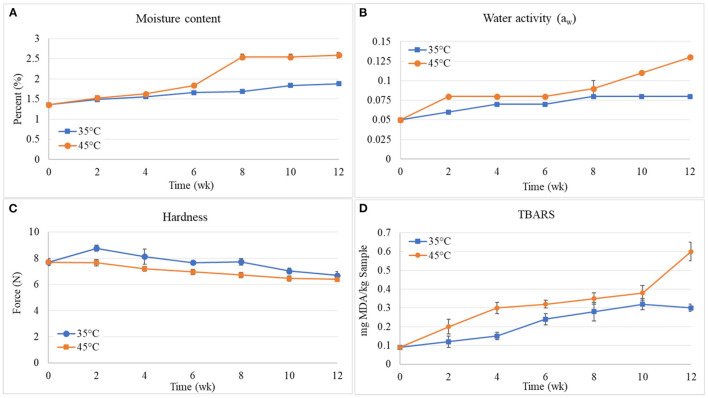
Moisture content **(A)**, water activity (*a*_w_) **(B)**, hardness **(C)**, and TBARS-value **(D)** of JC30 samples stored at 35 and 45°C.

Analysis of the shelf life of cracker JC_30_ was carried out once in 2 weeks for 12 weeks. The samples were packed in laminated aluminum foil bags and stored in two controlled conditions at temperatures of 35 and 45°C, respectively. The moisture content, water, activity (*a*_w_), hardness, and TBARS-value of JC30 samples stored at 35 and 45°C were measured and the results of moisture content, water activity, and hardness are given in Figure 3.

From Figure 3A, it is observed that the moisture content in jellyfish crackers increased with increasing the storage time for both crackers stored at 35 and 45°C. Furthermore, the moisture content in the crackers stored at 35°C is lower than those stored at 45°C. This result was correlated with that of Romeo et al.'s (46) study. This phenomenon was probably due to water migration from the environment (47). This kind of result proves that the storage conditions cause a difference in moisture content. The low moisture content of the products is important for prolonging their shelf life.

From Figure 3B, the water activity in jellyfish crackers increased with increasing the storage time for both crackers stored at 35 and 45°C. The results are in accordance with the moisture results in Figure 3A. Romeo et al. (46) observed the decrease in water activity was correlated to the moisture content in cookies and this decrease was less at 30°C compared to cookies at 20°C. During the first week, increase in water activity was observed ranging from 0.05 to 0.06 and 0.08 at 35 and 45°C, respectively. But during the second to sixth weeks, water activity gain for these samples was no marked change ranging from 0.06 to 0.07 and 0.08 to 0.09 at 35 and 45°C, respectively. In the last 3 weeks from 12 weeks of the storage time, the increase was huge ranging from 0.09 to 0.13 for crackers stored at 45°C. However, the water activity of crackers stored at 35°C was not marked changing from 0.07 to 0.08. When the crackers were stored at 35°C, the crackers took 3 months for an increase in water activity from 0.05 to 0.08, whereas the crackers stored at 45°C took only 2 weeks.

From Figure 3C, the crispiness as evaluated from the hardness value of jellyfish crackers showed that it tended to decrease with the increase in the storage time for both crackers stored at 35 and 45°C. The increase of the storage time increased the humidity (as shown in Figure 3A) and consequently caused a soft texture of the jellyfish cracker. This behavior was also reported by Morais et al. (48). The changes undergone by food hardness are directly linked to structural changes undergone during its storage. Water, due to its plasticizing effect, is one of the main elements responsible for these changes, thus lifting moisture was a consequent reduction of the cracker hardness. It may be due to the cracker absorbing the moisture from the atmosphere, which usually leads to softening of the cracker and the speed of moisture pick up is related to the ambient conditions (46).

In addition, the TBARS of crackers were also measured. Thiobarbituric acid reactive substances value is a measure of the formation of secondary oxidation products, for example, carbonyls, being responsible for the sensory impact of lipid oxidation. This assay measures MDA, which is a split product of an endoperoxide of unsaturated fatty acids resulting from the oxidation of lipid substrates. The MDA reacts with TBA forming a pink chromogen (TBARS), which is measured spectrophotometrically at 532–535 nm. A linear regression curve of the standard of TEP vs. the relative light unit for each standard was plotted. The TBARS as the milligram of MDA per kg of sample was calculated using the relative light unit obtained from the sample and equation generated by the standard curve. The results on the measurement of TBARS-values (expressed as absolute OD) as given in Figure 3D. It was found that TBARS-values of jellyfish crackers during storage at temperatures of 35 and 45°C were trended to increase as the storage time increased. The increase in TBARS-values during storage of jellyfish cracker is presented with an initial value of 0.09, the TBARS-values increased to 0.12, 0.15, 0.24, 0.28, 0.32, 0.30 and 0.20, 0.30, 0.32, 0.35, 0.38, 0.60 mg MDA/kg sample at 35 and 45°C, respectively after 12 weeks of storage. The TBARS-values of jellyfish crackers at the temperature of 45°C were higher than those of jellyfish crackers at the temperature of 35°C. This implies that with the increase in storage time and temperature, the formation/development of secondary oxidation products increases. Bunkar et al. (49) also reported an increase in TBARS-value of instant kheer mix powder during storage and this increase was found to be temperature dependent.

## Conclusions

The wheat flour was substituted with jellyfish to enhance the nutritional characteristics of crackers. The color and the texture of the crackers were affected by the jellyfish used as raw material. The crackers prepared with jellyfish had darker color and more brittle than those without jellyfish. Some adverse effects of substituted jellyfish on physical and sensory characteristics were observed. The texture of the enriched crackers is the important attribute that is criticized by jellyfish addition. The crackers with the substitution of jellyfish at levels of 30% obtained characteristics of cracker, liking scores, as well as the subjective quality of the final product. The quality of jellyfish crackers depends on storage temperature; however, jellyfish crackers had good physical and chemical conditions, being able to be consumed for 12 weeks stored at 35°C. Based on the findings, the production process of jellyfish crackers is cost-effective because the utilization of the jellyfish by-product could minimize the cost of waste disposal. The jellyfish crackers could be a remarkable source of protein, because of their high quality and cost.

## Data Availability Statement

The original contributions presented in the study are included in the article/supplementary material, further inquiries can be directed to the corresponding author/s.

## Ethics Statement

Ethical review and approval was not required for the study on human participants in accordance with the local legislation and institutional requirements. Written informed consent for participation was not required for this study in accordance with the national legislation and the institutional requirements.

## Author Contributions

SM and WS carried out the experiments, collected the data, analyzed the data, and wrote the manuscript. WP participated in data analysis and discussion of the results. PL participated in the revision of the manuscript. All authors contributed to the article and approved the submitted version.

## Funding

This work was financially supported by the Thailand Science Research and Innovation (TSRI) and the National Science and Technology Development Agency (NSTDA) (Grant No. RUG5950014).

## Conflict of Interest

The authors declare that the research was conducted in the absence of any commercial or financial relationships that could be construed as a potential conflict of interest.

## Publisher's Note

All claims expressed in this article are solely those of the authors and do not necessarily represent those of their affiliated organizations, or those of the publisher, the editors and the reviewers. Any product that may be evaluated in this article, or claim that may be made by its manufacturer, is not guaranteed or endorsed by the publisher.
